# Defining an onychomycosis cohort: The importance of mycologic sampling

**DOI:** 10.1016/j.jdin.2026.02.014

**Published:** 2026-03-20

**Authors:** Dustine Reich, Shari R. Lipner

**Affiliations:** a Weill Cornell Medicine, New York, New York; b Department of Dermatology, Weill Cornell Medicine, New York, New York

**Keywords:** antifungal, nail disease, nail fungal infection, onychomycosis, telemedicine

*To the Editor:* We read with interest Gupta et al’s retrospective cohort study of antifungal prescription pattern differences between telemedicine and in-person encounters for onychomycosis.^1^ The authors reported that patients evaluated by telemedicine versus in-person encounters for new onychomycosis diagnoses received fewer antifungal prescriptions. Herein, we emphasize the importance of mycologic sampling for onychomycosis diagnosis and suggest methodological improvements that would strengthen the validity of their findings.

Onychomycosis is the most common nail disorder seen in clinical practice, but many nail conditions share similar clinical features.^2^ Therefore, making a diagnosis of onychomycosis without laboratory confirmation is highly inaccurate.^2^ Notably, in Gupta’s database study, nail clippings with histopathology and potassium hydroxide with microscopy analyses could not be assessed.^1^ Thus, their onychomycosis diagnoses were not confirmed and could include a broad differential, such as psoriasis, mechanical onychodystrophy, or retronychia. For study conclusions to be meaningful, we suggest instead using a dataset with confirmatory data. For example, we performed a retrospective case-control study of 1256 patients with onychodystrophy who had nail clippings and histopathology performed and found that <50% of patients presenting with onychodystrophy had mycologically confirmed onychomycosis.^3^

Gupta et al’s primary finding was that the telemedicine versus in-person group was almost twice as likely not to be prescribed antifungals (odds ratio: 1.79, *P* < .001, E-value: 1.8).^1^ However, telemedicine workflows may differ among dermatologists. For example, in our specialty nail clinic, we require that all patients with suspected onychomycosis have an in-person visit for sampling, and if confirmed, antifungal medications are initiated during a follow-up telemedicine visit.^4^ Notably, in a retrospective study of 107 telemedicine visits, including 96 patients, we found that onychomycosis was the most common diagnosis for virtual follow-up visits (18/50, 36%). Our clinical experience is that telemedicine is appropriate for onychomycosis management, including prescribing antifungals, monitoring for efficacy and adverse events, and counseling on prevention of recurrence.^5^

Furthermore, Gupta et al reported statistical significance for differences in antifungal prescribing patterns for telemedicine versus in-person visits 3 months following the initial encounter.^1^ While we agree that it is important to follow patients with nail disorders longitudinally, based on Food and Drug Administration–approved courses for antifungals for onychomycosis and average nail growth rates, longer time intervals are generally needed to observe improvements.^2^ Therefore, it is expected that prescribing patterns would not change at the 3-month follow-up.

In sum, Gupta et al highlight important research questions regarding the role of telemedicine for the treatment of onychomycosis. We recommend that future studies incorporate mycologically confirmed onychomycosis cases so that findings are generalizable to clinical decision-making for antifungal prescribing and appropriate integration of telemedicine into dermatologic care.

### Declaration of generative AI and AI-assisted technologies in the writing process

Not applicable.

## Conflicts of interest

Dr Lipner has served as a consultant for Moberg Pharmaceuticals and BelleTorus Corporation. Dustine Reich has no conflicts of interest to declare.
